# The impact of the COVID-19 pandemic on UK parents’ attitudes towards routine childhood vaccines: A mixed-methods study

**DOI:** 10.1371/journal.pone.0306484

**Published:** 2024-08-13

**Authors:** Helen Skirrow, Celine Lewis, Habiba Haque, Lena Choundary-Salter, Kim Foley, Elizabeth Whittaker, Ceire Costelloe, Helen Bedford, Sonia Saxena

**Affiliations:** 1 School of Public Health, Imperial College London, London, United Kingdom; 2 Population, Policy and Practice, Great Ormond Street Institute of Child Health, University College London, London, United Kingdom; 3 London North Genomic Laboratory Hub, Great Ormond Street Hospital, London, United Kingdom; 4 The Mosaic Community Trust, London, United Kingdom; 5 Section of Paediatric Infectious Diseases, Imperial College London, London, United Kingdom; 6 Department of Paediatric Infectious Diseases, Imperial College Healthcare NHS Trust, London, United Kingdom; 7 Institute of Cancer Research, London, United Kingdom; Universitas Syiah Kuala, INDONESIA

## Abstract

**Background:**

COVID-19 vaccines were key to controlling the pandemic and vaccination has been discussed extensively by the media and the public since 2020. We aimed to explore parents’ attitudes towards routine childhood vaccination since COVID-19 and how the pandemic impacted their experiences of getting their child vaccinated.

**Methods:**

We used a mixed-methods approach—involving a questionnaire survey followed by focus groups. We partnered with The Mosaic Community Trust, an ethnic minority women’s group based in a deprived area of North-West London, United Kingdom (UK) with historically low childhood vaccine uptake. Descriptive findings from the questionnaires were reported and chi-square analyses performed to examine differences by ethnicity. Thematic analysis of the free-text questionnaire responses and focus groups was undertaken, guided by the COM-B model of Capability, Opportunity, and Motivation.

**Results:**

Between Jun-Oct 2022, 518 parents completed the questionnaire (25% from ethnic minorities). Between March-May 2023 we held four focus groups with 22 parents (45% from ethnic minorities). Most parents (>90%) thought routine childhood vaccines for children were important. Over a third (38%) of all parents reported having more questions about childhood vaccines since COVID-19, though among parents belonging to an ethnicity group other than white, 59% said they had more questions compared to those of any white ethnicity group (30%, (p = <0.0001)). Difficulties accessing vaccine appointments were commoner reasons for children’s vaccinations being delayed than parents increased concerns about vaccines. Since COVID-19 some parents felt vaccinations were even more important, and a very small minority felt the pandemic had made them mistrust vaccinations.

**Conclusion:**

Following COVID-19, we found parents remain confident in childhood vaccines. However, some parents, particularly from ethnic minority groups may have more questions about childhood vaccines than pre-pandemic. Post COVID-19, to address declining vaccine uptake, parents need easy access to healthcare professionals to answer questions about childhood vaccinations.

## Background

The United Kingdom’s (UK) longstanding universal and free childhood vaccination programme currently protects children by age 5 years against 14 infectious diseases, however the pandemic may have changed parents’ attitudes to vaccinating their child [1]. The UK’s COVID-19 vaccination programme commenced in December 2020 [2] and has been instrumental in controlling morbidity and mortality caused by the SARS CoV-2 virus [3]. The pandemic and vaccination programme have been widely covered by both mainstream [4] and social media [5], potentially exposing the public to a high volume of vaccine information [6]. The World Health Organization warned in 2020 that the world was facing not only a pandemic, but also an ‘infodemic’ through the widespread dissemination of medical and scientific information to the public, including misinformation [7]. There has been concerns that this overload of information could increase vaccine hesitancy [8]–defined as indecisiveness about a vaccination decision [9].

The COVID-19 pandemic caused a decline in routine childhood vaccination uptake globally [10], with disruption to routine vaccine services reported across 170 countries [10]. For example, a survey of Indonesian parents found that childhood vaccinations were delayed due to both vaccine services ceasing and parents choosing to delay vaccines during the pandemic [11]. In the UK the declines in childhood vaccine uptake have persisted past the initial stages of the pandemic [12–14]. In the UK, parents experienced difficulties accessing routine childhood vaccines during the first wave of the pandemic and confusion about whether routine vaccines were continuing [15, 16]. Pre-existing inequalities in childhood vaccine uptake between different ethnicity groups and among more disadvantaged areas were also exacerbated by the pandemic [17]. In the United States, parents’ attitudes to routine childhood vaccines were found to have been impacted by the pandemic [18], including some parents being more hesitant about vaccines recommended by healthcare workers [19].

In the UK, regular surveys conducted by public health bodies pre and post pandemic report high confidence in routine childhood vaccinations with the most recent (2022) survey reporting for example that 90% of parents *‘trust’* vaccines [20]. However, the 2022 United Kingdom’s Health Security Agency (UKHSA) survey recruited parents registered with the commercial marketing parent app ‘Bounty’ and under-represented parents from London and from ethnic minorities [20], groups who are less likely to have their children vaccinated [14, 21, 22]. As inequities in vaccine uptake among ethnic minorities appear to be widening, exploring perspectives about the impact of the pandemic on routine childhood vaccine attitudes among these groups is an urgent priority to inform interventions to address declining routine childhood vaccine uptake and address inequalities [14].

We partnered with The Mosaic Community Trust (Mosaic) [23, 24] who are based in a more deprived, ethnically diverse area of London with historically low childhood vaccine uptake–for example in the local area only 74% of 5 year olds have received the recommended two doses of measles, mumps and rubella (MMR) vaccines compared to 84% in England [14]. Using a mixed-methods approach, including a parental survey using a questionnaire followed by focus groups, we aimed to explore and describe how the COVID-19 pandemic had impacted parents’ attitudes towards, and experiences of, accessing routine childhood vaccinations. Given inequities in childhood vaccine uptake have increased since the pandemic in the UK [17], we also examined differences in responses between ethnic minority respondents and non-ethnic minority respondents.

## Methods

### Partnership with The Mosaic Community Trust

Our public partners The Mosaic Community Trust [23] were involved from the study’s start, building on a pre-existing collaboration [25]. The partnership between the study investigators and the Mosaic Community Trust involved shared power, reciprocity and respect throughout [26]. Mosaic is a community support group of around 100 ethnic minority, predominantly Asian or British Asian or Arabic women, the majority of whom are Muslim. Mosaic provides health and wellbeing support and links into local healthcare and mental health services through regular community meetings [23]. Mosaic named this study “Why has nobody asked us?!” to acknowledge that the views of ethnic minority parents living in an area of high deprivation with low vaccine uptake had not been previously considered. A mixed-methods approach was agreed on comprising a survey using a questionnaire followed by focus groups. In this paper we draw on data gathered from both the survey and focus groups on the impact of the COVID-19 pandemic on parents’ attitudes and experiences of routine childhood vaccines. These are part of the findings from the wider study aimed at investigating why some children in the UK are unvaccinated or vaccinated late.

### Survey

#### Questionnaire design

We co-developed a questionnaire based on brainstorming sessions with Mosaic and previous surveys of parental vaccine attitudes and hesitancy [27–30]. The COM-B model [31], previously applied to vaccination behaviour [30, 32, 33], was also used to guide topic development. The COM-B model suggests that ‘*Capability’ (*knowledge and skills), ‘*Opportunity’* (physical access & convenience, availability of information**)** and ‘*Motivation’* (Attitudes, values, emotions) are needed to perform a certain *‘Behaviour’* such as vaccination (B) [31]. Other stakeholders in North-West London such as the Maternity Champions, Community Champions, and the local public health department were also involved in informing question topics. Questions were developed, tested, and refined for acceptability and understanding among Mosaic members and other stakeholders. Some question topics were also considered to be better suited to be included in the subsequent focus groups to allow more in-depth exploration.

The questionnaire consisted of the following sections: 1) demographics, 2) routine vaccines for children in the UK 3) experiences of accessing information about routine vaccines for children in the UK and 4) routine vaccines for children in the UK and the COVID-19 pandemic. Demographic questions included age, ethnicity, religion, gender, geographical location, education, household composition and number of children. Respondents were asked a series of questions regarding their perceived importance of routine vaccination of their child/ren and whether they had ever delayed or refused a vaccine. For each discrete statement, respondents indicated their level of agreement/disagreement on a 7-point Likert scale between *Strongly disagree* to *Strongly agree*. There was also a free-text response option for respondents to make any other comments about routine vaccines recommended for children in the UK. They were then asked about information they received before their children’s vaccination and their experiences of accessing routine vaccine appointments for their children. Then there were questions about the impact of the COVID-19 pandemic on their views and experiences of childhood vaccines again using a Likert scale of agreement/disagreement. At the end of the questionnaire, respondents were provided with another opportunity to add any additional comments. The full questionnaire can be found in the S1 File in S1 Data.

#### Survey recruitment

Participants were recruited to the study through either through online promotion or by Mosaic who promoted the study in North-West London through local networks of parents and families. Mosaic promoted both online and paper questionnaire completion. Paper questionnaires were distributed in person by Mosaic Community Health workers to parents who attended the Mosaic community group meetings. Online promotion also involved a link to the questionnaire being emailed to community organisations including Maternity Champions, Maternity Voices Partnership, Community Champions, and Connecting Care for Children who were invited to share and promote it through their networks by email and social media. By approaching a range of community groups embedded among local peer support networks, our recruitment strategy sought to recruit particularly from grass roots organisations and therefore recruit underrepresented groups. The questionnaire was also advertised and promoted using Instagram paid for promotion which was geographically targeted to adults living North-West London. The questionnaire was also shared and distributed via the research team’s personal twitter accounts, including linking to other researchers and organisations interested in vaccine uptake.

Eligible participants were required to be aged 18 years or over, resident in the UK with children aged 10 years or younger at the time of completing the questionnaire. The questionnaire was live online and promoted from 7^th^ June 2022 until 6^th^ November 2022 though online promotion by Instagram was only between 12^th^ August– 28^th^ September 2022. The questionnaire was prefaced by an information page explaining the study, and how the data was to be used. Participants were informed that by taking part they agreed for their responses to be used for research purposes. Participants were required to confirm (by tick-box) at the start that they met the eligibility criteria and that they consented to participate in the study. Apart from the question confirming their eligibility, answers to the questions were optional. At the end of the questionnaire participants were invited to indicate their willingness to take part in a follow-up focus group by leaving their contact details.

### Focus groups

#### Recruitment and data collection

Respondents who included their details at the end of the questionnaire were contacted later and invited to take part in focus groups. They were given a choice of taking part in either in-person or online focus groups. Written informed consent was obtained from each participant. Depending on the preference of the participant, the consent form was sent and returned completed via email or in person. For the final fourth focus group participants were recruited from local parents among Mosaic’s network, as data saturation had not been reached by recruiting from the questionnaire respondents alone after three focus groups. Four focus groups were conducted in total, two in person at a community venue used by Mosaic for meetings in Westminster, North-West London and two online via Zoom. The topic guide (see S2 File in S1 Data) was informed by the questionnaire responses and included topics such as attitudes to vaccination, experiences of accessing vaccinations and how the pandemic had impacted participants’ views on routine childhood vaccination. Participants received a £10 gift voucher in recognition of their time and contribution. Focus groups were conducted between the 15^th^ March and 24^th^ May 2023 and lasted between 60 and 90 minutes. All the four focus groups, both online and in person, were conducted with both HS and HH acting as facilitators with HB, CL and EW acting as co-facilitators at different focus groups.

#### Questionnaire analysis

Descriptive statistics of discrete choice question responses are reported for all COVID-19 questions and the question on the importance of vaccinating children. Given the longstanding disparity in childhood vaccine uptake between different ethnicity groups [17, 21], Chi-square analyses were then conducted to test for significant associations between ethnicity and agreement/disagreement with the statements on how their attitudes to routine childhood vaccines had been impacted by the COVID-19 pandemic. Respondents were split into two ethnicity groups as we were underpowered to analyse by different ethnicity subgroups. Parents who answered which of the following best describes your ethnicity? as ‘*White*, *White Irish or Other White background’* were compared to those that answered any of ‘*Any Mixed background*, *Black or Black British or any Black background*, *Asian or British Asian or any Asian background*, *Chinese or other or prefer not to say’*. Likert responses were then also dichotomised *into ‘Agreed’ (Strongly Agree*, *Agree or Somewhat Agree)* and ‘*Disagreed’ (Neither Agree or Disagree*, *Somewhat Disagree*, *Disagree or Strongly Disagree)*. Parents who left the question about ethnicity blank were not included in the chi-square analysis.

#### Focus group analysis

Focus group recordings were transcribed verbatim by an external professional transcription service used by Imperial College London. Both the questionnaire free-text responses and the data collected in the focus groups were analysed using a codebook thematic analysis [34, 35], following an inductive and deductive coding scheme. Deductive codes were based on the COM-B model [31] as well as the study objectives and previous literature, while inductive codes were developed from the data. To enhance the rigour of the analysis, coding approaches and data interpretations were discussed between HH, HB, CL, and EW, with CL also double coding to check for agreement.

#### Ethical approval

Ethics approval was granted on 26^th^ January 2022 by the Imperial College Research Ethics Committee (study reference: 22IC7458).

## Results

### Questionnaire sample

A total of 518 parents completed the questionnaire, 476 online and 42 on paper (paper respondents). The total number of respondents answering each question, differs. Of the 50 paper questionnaires given to Mosaic community health workers to distribute, 42 were completed. The response rate of the online questionnaire is not known due to wide promotion, but the paid promotion feature on Instagram was used from 12^th^ August 2022 to 28^th^ September 2022 and reached 27,352 accounts and achieved 317 link click engagements. Characteristics of participants in the survey are shown in Table 1. Demographic characteristics between paper and online respondents differed. The average age of all respondents was 37 years (median and mean) and ranged between 19–57 years though paper respondents had a lower median age of 35 years. Among all respondents, 25% were from ethnic minority groups, though among paper respondents, the majority (83%, 35/42) were from ethnic minorities. The most common ethnicity among paper respondents was ‘Asian or British Asian’ (n = 17) and the 14 paper respondents who described themselves as ‘Other ethnicity group’ gave their ethnicity as ‘Arabic’. Among paper respondents the majority were Muslim (88%, n = 37/42). Only 31% (n = 13) of paper respondents were university educated with the majority completing secondary school education to either 16 years (29%, n = 12) or 18 years (26%, n = 11). Paper respondents also differed from online respondents with respect to family size: over a third of the paper respondents who answered the question had 3 or more children (55%, n = 15/42) compared with only 7% (n = 33) of on-line respondents. Very few respondents answered that they were living with extended family members, 7% (n = 35) though among paper respondents this was higher (17%, n = 7/42).

**Table 1:** Characteristics of questionnaire respondents.

z	Respondents (n)	%
**Gender**		
**Female**	456	88
**Male**	11	2
**Not given**	51	10
**Geographical location**		
**North-West London**	203	39
**All London**	330	64
**Outside London**	72	14
**Not given**	116	22
**Country of birth**		
**United Kingdom (UK)**	267	51
**Non-UK**	149	29
**Not given**	102	20
**Ethnicity**		
**White or White British**	320	62
**Mixed**	28	5
**Black or Black British**	21	4
**Asian or Asian British**	43	9
**Other, ethnic group**	38	7
**Not given**	68	13
**Religion**		
**No religion**	219	42
**Christian**	164	32
**Muslim**	71	14
**Hindu**	8	2
**Jewish**	8	2
**Not given**	48	9
**Education**		
**Educated to primary school level, secondary school up to 16 years or 18 years**	48	9
**Educated beyond 18 years at college, university or to post-graduate level**	415	80
**Not given**	55	11
**Household structure**		
**Raising children with partner**	428	83
**Single parent or other**	47	9
**Not given**	43	8
**Number of children**		
**1**	206	40
**2**	206	40
**3 or more**	48	9
**Not given**	58	11

Demographic characteristics of both paper (n = 42) and online (n = 476) questionnaire respondents (total n = 518).

### Focus group sample

In total 19% (n = 100) of all questionnaire respondents provided their details to be contacted for a follow-up focus group and were contacted by email by HS, or by phone by HH, to participate. Twenty-two responded to the invitation, with 16 taking part in one of the first three focus groups. The remaining six parents opted out prior to the focus groups. Mosaic recruited a further six women to take part in the final focus group from among the local parents who use Mosaic services but who had not completed the questionnaire. The characteristics of focus groups participants are outlined in Table 2, (100% were women) of whom 10 were from ethnic minority groups. The focus groups included parents who had chosen to delay or decline some childhood vaccinations (see Table 2).

**Table 2:** Characteristics of focus group participants.

No	Ethnicity	Online or in person focus group in North-West London	Vaccines ever delayed or refused for the child/ren (not including childhood Covid-19 vaccine)
**1**	White (other)	In person (FG1)	None
**2**	White (British)	In person (FG1)	Flu, whooping cough & additional polio booster
**3**	Asian or Asian British	In person (FG1)	None
**4**	Asian or Asian British	In person (FG1)	None
**5**	Other ethnic group (Arab)	In person (FG1)	MMR
**6**	White (British)	Online (FG2)	None
**7**	White (British)	Online (FG2)	Paid for some vaccines separately.
**8**	Mixed	Online (FG2)	None
**9**	White (Other)	Online (FG2)	None
**10**	White (Other)	Online (FG2)	Delayed MMR and paid privately for other vaccinations to be spaced out.
**11**	White (British)	Online (FG2)	None
**12**	White (British)	Online (FG2)	None
**13**	White (British)	Online (FG2)	None but chose to have 1 year old vaccines at separate appointments.
**14**	White (Other)	Online (FG2)	Preschool booster
**15**	Asian or Asian British	Online (FG2)	MMR—both 1 year and preschool booster declined.
**16**	White (British)	Online (FG2)	None
**17**	White (British)	In person (FG2)	None
**18**	Asian or Asian British	In person (FG2)	MMR vaccine
**19**	Asian or Asian British	In person (FG2)	None
**20**	Asian or Asian British	In person (FG2)	None
**21**	Other ethnic group (Arab)	In person (FG2)	MMR vaccine

### COM-B model

**Fig 1:**
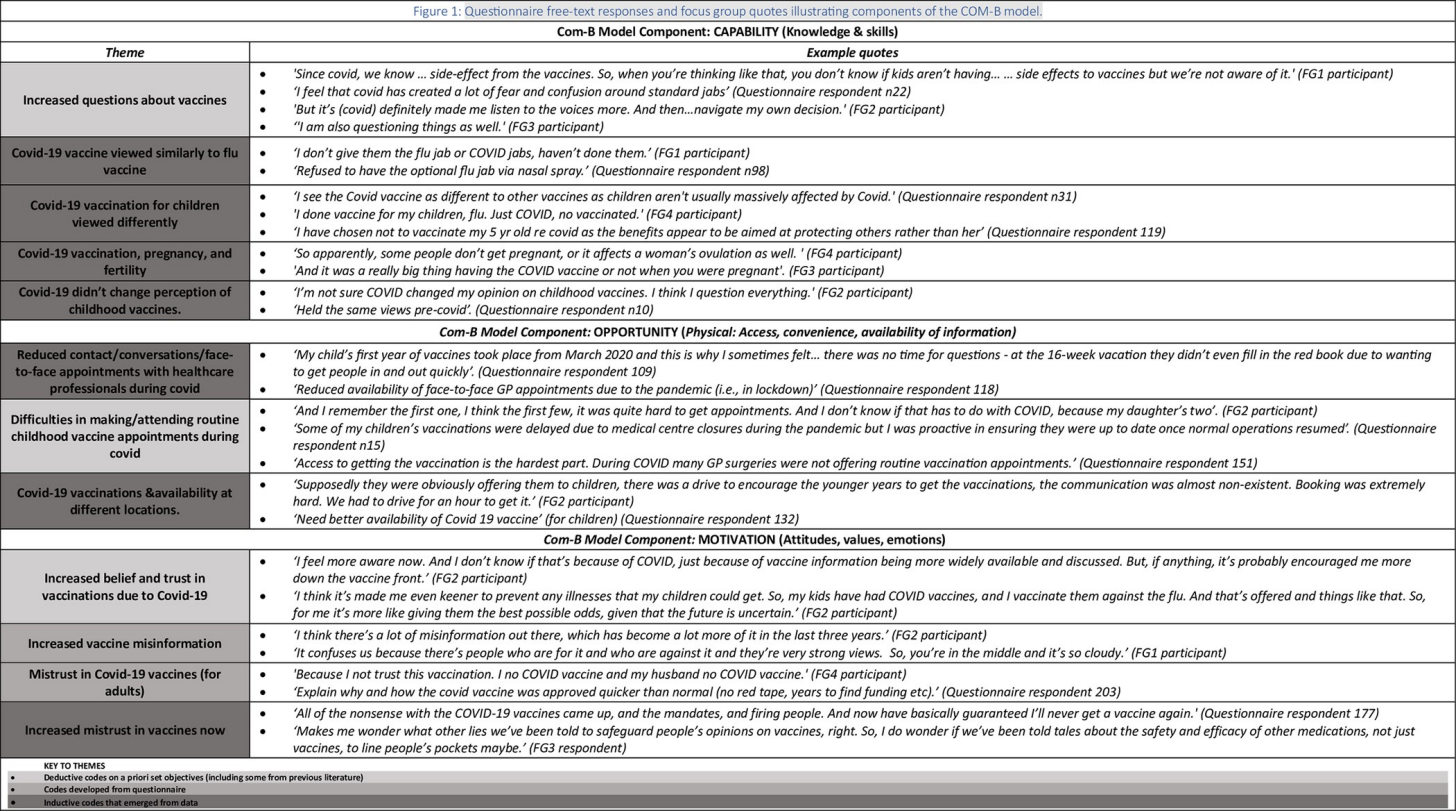
Example quotes from the questionnaire free-text responses and focus groups illustrating different themes of the COM-B model.

The following sections present data according to the COM-B model [31], which was used as guide for analysing how the COVID-19 pandemic has impacted routine childhood attitudes and experiences (see Fig 1). The themes draw on a combination of discrete question responses from the questionnaire, free-text questionnaire answers and data from the focus groups (see S3 File in S1 Data for number of questionnaire free-text responses). Given the variation in the number of respondents per question descriptive statistics and chi square analysis report on the percentages among those who chose to answer each question. Quotes from focus group participants and free-text responses are provided in Fig 1 to illustrate the key themes. Themes were developed from either deductive codes based on the a priori study objectives and some were inductive either developed from the questionnaire responses or from the focus group data. For example, attitudes specifically towards COVID-19 vaccination for children or adults were not explored but many free-text responses and focus groups participants discussed COVID-19 vaccines so were included as inductive themes.

### Capability

#### Knowledge and skills

Over a half (52%) of respondents agreed to some extent that their knowledge about routine vaccines had been increased by the pandemic (S4 File in S1 Data). However, just over a quarter (26%) disagreed, with the remainder (23%) neither agreeing nor disagreeing (S4 File in S1 Data). There was no difference between responses by ethnicity (S5 File in S1 Data).

#### Increased questions about vaccines

**Fig 2:**
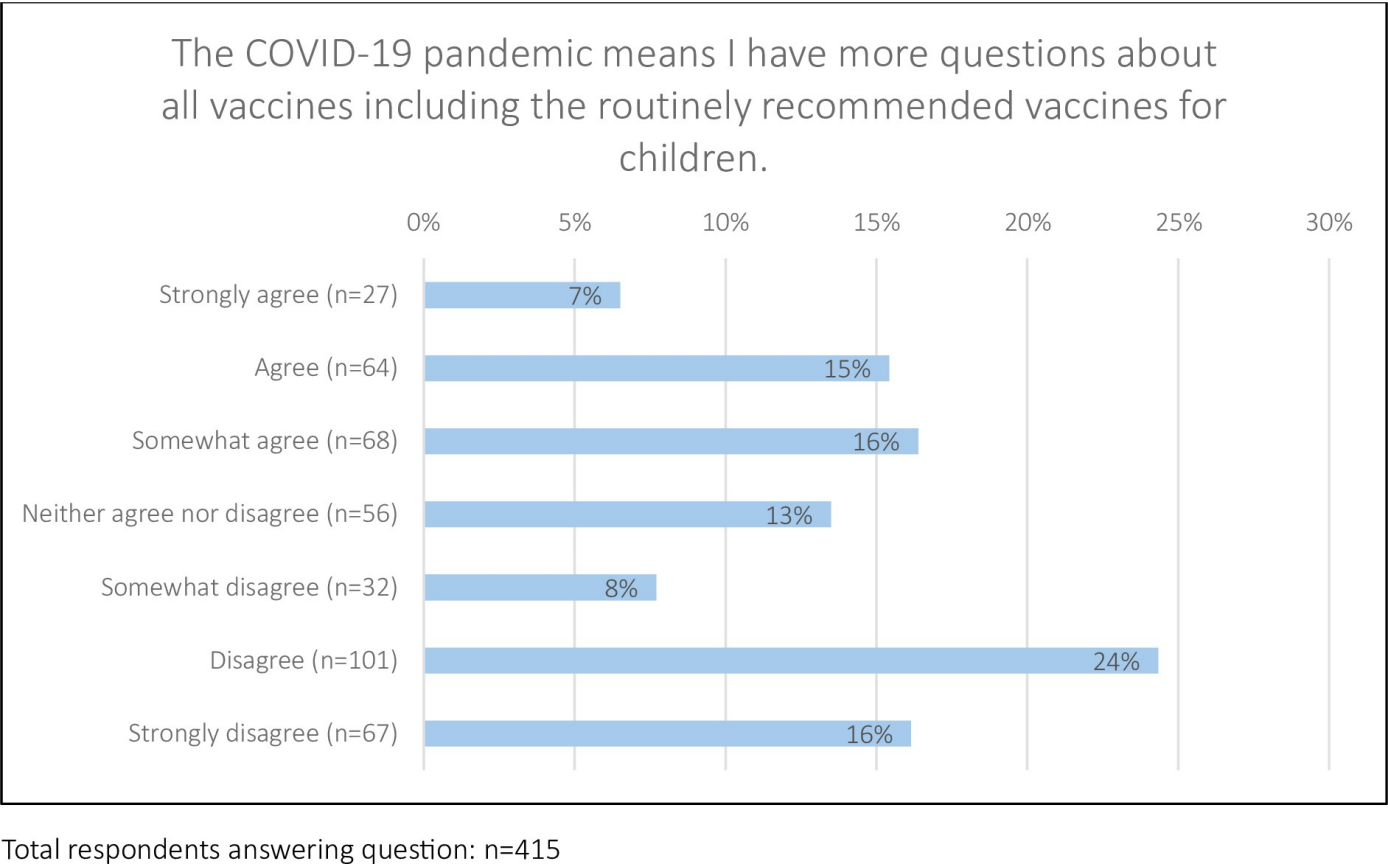
Respondents level of agreement/disagreement on a 7-point Likert scale between Strongly disagree to Strongly agree with the statement: ‘The COVID-19 pandemic means I have more questions about all vaccines including the routinely recommended vaccines for children’.

Over a third of questionnaire respondents (38%, n = 159/415) agreed to some degree that they had more questions about routine childhood vaccines since COVID-19 (Fig 2). Parents who described their ethnicity as “*Any Mixed background*, *Black or Black British or any Black background*, *Asian or British Asian or any Asian background*, *Chinese or Other or Prefer not to say’* were more likely to have questions following COVID-19–59% (n = 71/120) agreed that they had more questions since the pandemic about routine childhood vaccines compared to 30% (n = 84/281) of parents who identified as white ethnicity (p<0.0001, S5 File in S1 Data). Among paper respondents 63% (n = 26/41) agreed to some extent that the pandemic had increased their questions about routine vaccines compared to only 36% (n = 133/374) of online respondents.

In the focus groups some parents reported having more questions about routine childhood vaccines since the COVID-19 pandemic (Fig 1). Parents might now see vaccination as a choice compared with before the pandemic when they viewed it as something they did automatically for their children—*‘I think the impact of Covid*, *for me*, *has made me think that vaccinations are a choice*. *So*, *you’re actively choosing to do something*, *or you’re choosing not to do something*. *And it’s made it less compulsory and more voluntary’ (Focus Group (FG) 2 participant)*.

#### COVID-19 vaccine viewed similarly to flu vaccine

Although not specifically questioned about COVID-19 vaccines for their children, several questionnaire respondents and focus group participants discussed this and in doing so drew parallels with the childhood nasal flu vaccine (Fig 1). Both were seen as additional or optional by some parents, and not viewed as being part of the routine childhood vaccination offered in the UK. For example, one questionnaire respondent *(questionnaire respondent n122)* commented ‘*‘I chose not to get my son the flu vaccine or the current Covid vaccine*. *I feel both are not essential*.*’* Another parent felt that getting the flu vaccine during the pandemic was not needed ‘*Getting flu nasal was an unnecessary outing during lockdown and risks from it are minimal compared to other illnesses vaccines are offered for’ (questionnaire respondent n40)*.

#### COVID-19 vaccination for children viewed differently

Several parents mentioned their attitudes to COVID-19 vaccination for their children in either the questionnaire’s free-text responses or during the focus groups. COVID-19 vaccination was not viewed as important or necessary for children compared to adults, and parents often cited less severe symptoms of COVID-19 in children compared to adults as their rationale for not wanting the vaccine for their children (Fig 1). For example, one parent reported, *‘The Covid 19 jab was never needed for children as children were not even getting bad symptoms so why did they need a vaccine for covid if it didn’t even affect them much in the first place*?*’ (Questionnaire respondent n26)*.

#### COVID-19 vaccine and pregnancy and fertility

In the survey, a few parents expressed their own or other parents in their circle concerns about COVID-19 vaccines impacting on fertility; this issue was also raised in the focus groups (Fig 1). Additionally, in the focus groups, several participants mentioned their confusion about whether to have the COVID-19 vaccines during pregnancy. For example, one focus group participant said: *’Had both COVID vaccines whilst I was pregnant*, *which was a huge decision for me*. *Because at the time*, *they were like we don’t know what it’s going to do to you if you’re pregnant*.*’ (FG3 participant)*. Another commented on midwives not recommending COVID-19 vaccine strongly enough for pregnant women ‘*most midwives didn’t advocate strongly enough for pregnant women to have the covid vaccine’ (Questionnaire respondent n24)*.

#### COVID-19 did not change perception of childhood vaccines

Several respondents to the online questionnaire commented that the pandemic had no impact on their views and attitudes to childhood vaccines and that they had the same beliefs as pre-pandemic (Fig 1). For example, one questionnaire respondent wrote directly after the COVID-19 questions: ‘*Hasn’t changed my view’ (Questionnaire respondent n3)*.

Discrete choice answers indicated that most respondents felt that the pandemic had made them think it was more important for children to receive the routine vaccines (total 62% (n = 250/405) either strongly agreed, agreed, or somewhat agreed–S6 File in S1 Data) with only a minority of total respondents (13%, n = 57/405) disagreeing to any extent with this statement (S6 File in S1 Data). There was no difference between the different ethnicity groups about whether the pandemic had made them think routine vaccines were more important (S5 File in S1 Data).

### Opportunity

#### Reduced contact/conversations/face-to-face appointments with healthcare professionals during COVID-19

Questionnaire respondents and focus group participants both mentioned that the reduction in face-to-face appointments with different healthcare professionals during the pandemic–including midwives, health visitors and general practice staff—had given them fewer opportunities than they would have liked, to ask questions about routine childhood vaccines (Fig 1). *‘I had my baby during the end of COVID*, *so—so I felt like I didn’t get that much information and I was left out*.*’ (FG1 participant)*. That parent went on to describe the questions they wanted answering including: *‘There’s so many vaccines and I feel like it’s too much…*. *Why does a baby need to have all of these*?*’ (FG1 participant)*.

#### Difficulties in making/attending routine childhood vaccine appointments during pandemic

In the free-text questionnaire responses, many respondents reported their children’s routine vaccines had been delayed due to COVID-19 and finding it difficult to either schedule or attend an appointment (Fig 1). Access difficulties during COVID-19 were more frequently cited as a reason for a delayed vaccination than choosing to delay the vaccine because the pandemic made them question/doubt vaccines. For example, one parent commented: *‘For me personally in the height of the pandemic my GP services made it difficult to book any appointments so there was a lot of confusion on whether I could book vaccinations*, *my children missed the vaccinations they were due to have during the lockdown’* (*questionnaire respondent n28)*.

The majority (47% n = 194/414) strongly disagreed that following COVID-19 they were more likely to refuse or delay a vaccine for their child (S7 File in S1 Data), with a further 32% (n = 134/414) disagreeing or somewhat disagreeing and only 13% (n = 55/414) agreeing (S7 File in S1 Data). Similarly, 81% (n = 319/394) disagreed to some degree that the pandemic had made them refuse or delay a vaccine for their child (S8 File in S1 Data). Chi-square analyses however suggested that parents who answered that they belonged to an ethnicity group other than White ethnicity were more likely to refuse a vaccine (p = <0.0001) or had actually refused a vaccine for their child since the pandemic (p = 0.0031) compared to parents in the White ethnicity group (S5 File in S1 Data).

#### COVID-19 vaccinations and availability at different locations

Several parents reported via the free-text comments that although they wanted their children to have the COVID-19 vaccine, they had struggled to get an appointment. This was also raised in the focus groups where parents discussed the lack of availability of COVID-19 vaccines as well as not wanting to have routine childhood vaccines in some of the locations where COVID-19 vaccines had been offered (Fig 1). For example, ‘*With covid*, *…we all get jabbed by some randomest people in some random locations*. *We had ours in Westminster Abbey*. *Nice location but I wouldn’t take my child for his first vaccination there’ (FG1 participant)*. This was generally perceived to be because they would rather go to a healthcare setting, including pharmacies, with a trusted healthcare professional giving their children’s vaccines. For example, one parent commented ‘*it’s reassuring that when you’re at the GP you have it*, *because then you can ask any questions that you think of there and then*…*I remember having my Covid vaccine in a leisure centre and*, *yes*, *I don’t know that I would want to take (them) to a leisure centre to… not somebody that I know that’s vaccinating him either*. *Whereas even if it’s a nurse that I’m not familiar with at the GP surgery*, *at least this is my GP surgery that I go to*, *they reassure me*, *I ask them questions*. *I’ve got that bit of a relationship’ (FG3 participant)*.

### Motivation

#### Attitudes, values, emotions

In the survey, the overwhelming majority of parents (91%, n = 416/459) either strongly agreed or agreed that it is important to get their children vaccinated (S9 File in S1 Data). Including parents who somewhat agreed with this statement, increases this percentage to 95% (n = 435/459). There was a small (<10%, p = 0.0017) difference between the percentage of parents who agreed that it was important to get their child vaccinated who belonged to ethnicity groups other than White ethnicity compared to those who said they were White ethnicity (S5 File in S1 Data).

#### Increased belief and trust in vaccinations due to COVID-19

Some parents also discussed how the pandemic had made them believe vaccinations were more important than they had previously thought and that getting their children vaccinated was an even greater priority (Fig 1). This is linked to the theme about the pandemic having not changed their mind about vaccines–one parent commented after the COVID-19 section of the questionnaire *‘I was already a strong proponent for vaccines’ (questionnaire respondent 4)*.

Most respondents (66%) somewhat disagreed, disagreed, or strongly disagreed that the pandemic had made them question the safety of routine vaccinations for children (S10 File in S1 Data) though 28% somewhat agreed, agreed, or strongly agreed that it had. There were some differences between paper and online responses to this question with more paper respondents, 63% (n = 26/41) agreeing to some degree that the pandemic had made them question the safety of routine vaccines compared to only 24% of online respondents (n = 89/374). Chi-square analyses suggests that parents who identified as ethnicity groups other than White ethnicity were more likely to question routine vaccine safety post covid (51% n = 61/120) compared to those of White ethnicity ((18%, n = 50/330), p value = <0.0001, S5 File in S1 Data). Qualitative data revealed concerns such as about side effects to explain why vaccine safety has been questioned (see Fig 1).

#### Increased vaccine misinformation

In both the free-text questionnaire responses and the focus groups, parents discussed seeing an increase in vaccine misinformation and feeling that they had to try and make sense of this post-covid (Fig 1). For example, a parent discussed the impact of social media information on their ideas about vaccines during Covid ‘*media leave us to change our ideas about vaccinations in Covid…Because when I heard a lot of Misinformation or right information or wrong information from the media…but because in the phone media everywhere…Facebook*. *So now we…Confused*. *Yes’ (FG4 participant)*. Parents cited ‘*misinformation on the internet…*. *which is then repeated and shared among some peer groups’* as being a reason for some people not agreeing with vaccines *(questionnaire respondent n71)*.

#### Mistrust in COVID-19 vaccines for adults

In the focus groups, particularly the in-person ones, participants discussed actively declining COVID-19 vaccines for themselves and their wider family due to scepticism about COVID-19 vaccines for adults. Some questionnaire respondents mentioned similar feelings. This was despite no specific questions about COVID-19 vaccines for adults or children. They reported not trusting COVID-19 vaccines as they were ‘new’ and been developed quickly. For example, one questionnaire respondent said they are *‘afraid of any new vaccines including covid 19*. *So*, *we didn’t have it*. *Nobody knows it’s long-term effect’ (questionnaire respondent 240)*. There was also a sentiment expressed by some that they regretted getting the COVID-19 vaccines for example *‘Since the pandemic we hear so much about the effects of vaccines*. *I wish I hadn’t got the covid vaccine’*. *(Questionnaire respondent n330)*.

#### Increased mistrust in vaccines now

A very small minority of focus group participants and a few questionnaire respondents discussed the COVID-19 pandemic having increased their mistrust towards all vaccines (and not just specifically routine childhood ones), (see Fig 1). They cited mistrust in pharmaceutical companies and vaccination being promoted for financial gain for example ‘*There is a loud silence about conflict of interests of those flag bearing for vaccine production and their financial gain*. *The research and results done is/was minimal especially during the Covid era and very questionable’ (Questionnaire respondent 138)*.

## Discussion

### Main findings

In this mixed-methods study, we explored how parents’ attitudes towards, and experiences of getting routine childhood vaccinations have been impacted by the COVID-19 pandemic. We used the COM-B model [31, 33] to identify how different aspects of parents’ views and experiences have been affected. This provides insights into where action may be needed to tackle the downward trend in childhood vaccine uptake [14] in England, which started prior to the pandemic and has worsened since [12, 36, 37].

We found that post pandemic the majority (over 90%) of parents continue to consider routine childhood vaccination to be important–in keeping with studies conducted pre-pandemic [27, 28], in the early phase of the pandemic in 2020 [15] and subsequently [20]. A smaller majority (62%) reported feeling the pandemic had made them think childhood vaccination was even more important. However, we also found that while most parents still have confidence in routine childhood vaccines, some may have more questions about vaccines post COVID-19; over a third of parents reported that they had more questions post-pandemic. To our knowledge this is the first UK study conducted since the pandemic to examine specifically its impact on attitudes and to find that parents may have more questions. Some parents felt the pandemic had made them question the safety of routine childhood vaccines, though the majority (66%) did not. A very small minority of parents (<10%) reported that the pandemic had made them more likely to refuse or delay a vaccine, and again a very small minority of parents reported in the qualitative findings that their mistrust in all vaccinations has increased since the pandemic. Our findings are broadly in keeping with studies in the United States that find while parental vaccine confidence remains high, vaccine hesitancy, defined as indecisiveness about vaccination [9], may have increased since COVID-19 [19].

In keeping with previous studies, including one conducted during the first COVID-19 lockdown in 2020 [15, 16], parents reported finding the pandemic had made booking and attending vaccination appointments harder. Parents reported that these difficulties were more common reasons for their children not to be vaccinated or vaccinated late during or since the pandemic than COVID-19 prompting concerns about routine childhood vaccination. Our findings provide further evidence that parents in the UK have confidence in vaccination, and that anti-vaccination sentiment is not the main driver [38] of parents delaying or not vaccinating their children, even since COVID-19.

Though we were underpowered to report differences between different ethnicity sub-groups we found that parents who identified as belonging to ethnicity groups other than white may have more questions about childhood vaccines since the pandemic. More parents who completed the questionnaire on paper were from ethnic minorities (83% compared to 25% of all respondents), which may explain why 63% of paper respondents agreed to some extent that they had more questions since the pandemic compared to only 36% of online respondents. This difference in ethnicity between the online and paper respondents may also explain the differences with regards to other questions, such as more (63%) paper respondents reporting that the pandemic had made them question the safety of routine childhood vaccines compared to online respondents (24%). In the UK children belonging to ethnic minorities are also less likely to be vaccinated and these disparities have only worsened since COVID-19 [17]. During COVID-19 parents from ethnic minorities were also more likely to report confusion about whether routine childhood vaccine services were running [15] or to report difficulties accessing vaccine appointments [16].

Religion, ethnicity and deprivation have been found to intersect with regards to childhood vaccination attitudes and access in the UK [22, 27, 39]. For example, Muslim parents in the UK may refuse the childhood nasal influenza vaccine due to concerns about porcine gelatine content [40]. Though studies in predominantly Muslim countries have found that vaccine acceptance among Muslim populations can be high–for example over 85% of adults surveyed in an Indonesian sample would accept COVID-19 vaccines [41], so making associations between vaccine acceptance and religion requires caution. Over 80% of the paper respondents to our survey were from ethnic minorities and gave their religion as Muslim, of whom 63% thought the pandemic had made them more likely to question the safety of routine childhood vaccines compared to 24% of online respondents.

Future research could consider how local religious networks can be utilised to increase childhood vaccine uptake among predominantly Muslim communities such as where the Mosaic Community Trust is based. For example, targeted interventions to address attitudes and access to childhood vaccinations among UK orthodox Jewish families who have low vaccine uptake have been previously developed [30]. To address declining vaccine uptake tailored interventions and resource are needed which take into account the multiple dimensions of local religious, cultural and ethnicity networks in areas of low vaccine uptake.

Despite our questionnaire not asking specifically about COVID-19 vaccines for children, or adults, many parents volunteered their views. This is unsurprising given the questionnaire becoming live in 2022 which coincided with a recent non-urgent recommendation of COVID-19 vaccines for 5–11 year olds by the UK’s expert body on vaccination [42]. Some parents viewed COVID-19 vaccines for children as non-essential, and some in a similar way to the childhood nasal flu vaccine and therefore ‘different’ from other routine childhood vaccines. Some parents expressed negative views towards COVID-19 vaccines for children while some, particularly those who completed the online questionnaire, felt that the UK should have recommended COVID-19 vaccines for children earlier as other countries such as the United States had done [43]. Other research has found that parents may be less willing to accept COVID-19 vaccines for their children compared to themselves [44, 45] and in our research this was primarily due to COVID-19 vaccines being seen as ‘non-essential’ for children. A study in the USA found that parents’ main reason for not wanting to vaccinate their children against COVID-19 was due to concerns about vaccine safety and side-effects [46]. A minority of parents we surveyed also discussed feelings of mistrust towards COVID-19 vaccines for adults or concerns about the safety of COVID-19 vaccines for pregnant women or impacts on fertility. Uncertainty and confusion around the advisability of COVID-19 vaccines for women of reproductive age are in keeping with previous research and highlights the importance of clear, timely, tailored communication around vaccination, pregnancy, and fertility [47, 48]. In previous work we have also found that uptake of pertussis vaccine in pregnancy and subsequent childhood vaccine uptake of MMR vaccines are related [49] so it is perhaps unsurprising that in a survey of childhood vaccine attitudes that themes related to vaccination and pregnancy emerged.

### Strengths and limitations

The main strength of this study is that partnering with Mosaic enabled targeted recruitment in an area of North-West London with a historically low vaccine uptake, with a quarter of respondents to the survey from ethnic minority groups. The focus groups also consisted of 45% of women from ethnic minorities. This is compared to previous parental surveys/studies that have often included fewer parents from ethnic minorities [15, 27], or from London which has lower vaccine uptake [20]. Our mixed-methods approach using a questionnaire survey followed by the focus groups also allowed greater insight into parents’ attitudes to vaccination since the pandemic.

However, there are limitations including varying numbers of parents completing each question and small numbers of parents from different ethnicity sub-groups. This means we are unable to report on differences in attitudes between different ethnicity sub-groups limiting our conclusions about different ethnicities’ attitudes towards vaccination since the pandemic. This aggregation of non-white ethnicity into one group is a significant limitation and fails to appreciate the heterogenicity of groups classified as ethnic minorities in the UK and the multi-dimensional concept of ethnicity [50]. Future research should seek to unpick variation between different ethnic minorities groups attitudes and experiences of childhood vaccination and how interventions can be tailored to address inequalities.

The parents we surveyed were not asked their attitudes pre-pandemic therefore another limitation is that we did not directly compare the impact of the pandemic on the study participants individual vaccine attitudes. Also due to eligibility the parents questioned included both those who had children pre-pandemic, whose pre-existing attitudes may have been changed by the pandemic, and also parents who had children during the pandemic, whose vaccine attitudes may have been influenced by giving birth during COVID-19. The questionnaire was live in 2022 and the focus groups took place in 2023 so parents may also have inaccurately recalled the impact of the pandemic on their access to vaccinations. However, researching attitudes two years after the start of the pandemic enabled its longer-term implications on routine vaccine attitudes to be explored.

### Implications for policy and practice

Measles outbreaks in London in 2023 [51] and the risk of increasing infectious outbreaks due to England’s declining childhood vaccine uptake [14] are a public health concern [37]. Our findings suggest that since the pandemic, parents need easier access to healthcare professionals to answer their questions about routine vaccines. This is in keeping with UKHSA’s parents’ survey in 2022 that found over a third of parents wanted more information before their child’s vaccine appointment, though the nature of the information wanted is unclear [20]. Conversations with healthcare professionals are important influences on vaccine decisions [52] and all staff who see parents and families should be trained in how to answer parents’ questions about vaccines. Facilitating parents’ easier access to healthcare professionals to answer their questions will need greater resourcing and staffing in primary care. This may be even more important in more deprived areas of the country with lower vaccine uptake and less existing resource [53], and also among ethnic minority parents.

## Conclusion

Our findings suggest that although parents remain confident in routine childhood vaccination, since the pandemic they want more information and may have more questions which need addressing before their child’s vaccine appointment. This maybe particularly the case for parents who belong to ethnic minority groups. Healthcare professionals need time and training to be able to confidently answer parents’ questions about routine vaccinations to address declining vaccine uptake in the UK. Providing parents with additional information about vaccination could help families have greater awareness about the importance of vaccination for protecting against infectious diseases throughout life. Interventions post COVID-19 to increase vaccine uptake should be locally tailored and incorporate co-design methodologies.

## Supporting information

S1 Data S1 File: Questionnaire, S2 File: Focus Group Topic guide, S3 File: Number of freetext responses to questionnaire, S4 File: COVID-19 and knowledge about routine vaccines recommended for children, S5 File: Impact of COVID-19 on parents’ attitudes to routine childhood vaccinations by Ethnicity, S6 File: COVID-19 pandemic and the importance of routine vaccines, S7 File: COVID-10 pandemic and likelihood of refusing or delaying routine vaccines, S8 File: COVID-10 pandemic and having refused or delayed routine vaccines, S9 File: Importance of getting children vaccinated, S10 File: COVID-19 pandemic and safety of routine vaccines.
(DOCX)
