# Supplementary material for: The impact of the COVID-19 pandemic on UK parents’ attitudes towards routine childhood vaccines: A mixed-methods study
Source: PLoS One. 2024 Aug 13;19(8):e0306484. doi: 10.1371/journal.pone.0306484 (PMC11321586; doi:10.1371/journal.pone.0306484)
Supplement: S1 Data — S1 File: Questionnaire, S2 File: Focus Group Topic guide, S3 File: Number of freetext responses to questionnaire, S4 File: COVID-19 and knowledge about routine vaccines recommended for children, S5 File: Impact of COVID-19 on parents’ attitudes to routine childhood vaccinations by Ethnicity, S6 File: COVID-19 pandemic and the importance of routine vaccines, S7 File: COVID-10 pandemic and likelihood of refusing or delaying routine vaccines, S8 File: COVID-10 pandemic and having refused or delayed routine vaccines, S9 File: Importance of getting children vaccinated, S10 File: COVID-19 pandemic and safety of routine vaccines. (DOCX) [file pone.0306484.s001.docx]

# Supporting Information

[Supporting Information 1](#_Toc164325628)

[Supporting 1: Questionnaire 2](#_Toc164325629)

[Supporting 2: Focus Group Topic Guide 14](#_Toc164325630)

[Supporting 3: Number of freetext responses to questionnaire 15](#_Toc164325631)

[Supporting 4: Covid-19 and knowledge about routine vaccines recommended for children. 16](#_Toc164325632)

[Supporting 5: Impact of Covid-19 on parents’ attitudes to routine childhood vaccinations by Ethnicity 17](#_Toc164325633)

[Supporting 6: Covid-19 pandemic and the importance of routine vaccines 18](#_Toc164325634)

[Supporting 7: Covid-19 pandemic and likelihood of refusing or delaying routine vaccines. 19](#_Toc164325635)

[Supporting 8: Covid-19 pandemic and having refused or delayed routine vaccines. 20](#_Toc164325636)

[Supporting 9: Importance of getting children vaccinated. 20](#_Toc164325637)

[Supporting 10: Covid-19 pandemic and safety of routine vaccines 21](#_Toc164325638)

# Supporting 1: Questionnaire

’Why did nobody ask us?!’

Understanding why parents and families think some children get vaccinated late or miss out on some of their routine recommended childhood vaccines.

Section 1: This section gives you some information about the questionnaire

Principal Investigator: Dr Helen Skirrow. Collaborator: The Mosaic Community Trust.

Co-investigators: Prof Helen Bedford, Dr Ceire Costelloe, Dr Celine Lewis, Dr Liz Whittaker, and Prof Sonia Saxena.

You are being invited to take part in a study to investigate why parents and families think some children are not being vaccinated or get vaccinated late. The information you give answering this study questionnaire could help improve how childhood vaccines are delivered. This could help more children get vaccinated, and on time, to protect them against potentially dangerous infectious diseases.

The study is part of Dr Helen Skirrow’s PhD researching missed and late childhood vaccination in the UK. This is funded by the National Institute of Health Research of the Department of Health and Social Care. Helen is collaborating with The Mosaic Community Trust, a women’s community group in Westminster, London for this research. They have been involved in designing the questions.

You are eligible to participate in this questionnaire if you:

• live in the United Kingdom

• are aged 18 years or older

• are the parent or guardian of a child or children aged 10 years or younger

It is up to you to decide whether or not to take part.

If you do decide to take part by completing this questionnaire, you are still free to pull out at any time and without giving a reason.

The questionnaire will take around 10-15 minutes to complete.

You do not need to answer all the questions if you do not want to.

The Head of the Department of Primary Care and Public Health and the Research Governance and Integrity Team have approved this study.

- *At the end of the questionnaire you will be given the opportunity to give more information about your views on childhood vaccination by taking part in a focus group.*
- *The focus groups will be held online or if you live close enough, at a location in Westminster, North-West London depending on Covid-19 restrictions. Focus groups will be run by The Mosaic Community Trust and Dr Helen Skirrow.*
- *If you decide you would like to take part in a focus group you will be asked to provide your contact details for us to get in touch with you. In this case, the contact details you provide will not be linked to any of the questionnaire answers that you provide.*
- *For more information about this research please feel free to contact me (Dr Helen Skirrow) at any time and I will be happy to answer your questions. You can email me:* [*h.skirrow@imperial.ac.uk*](mailto:h.skirrow@imperial.ac.uk)

**Thank you for taking part in research if you do decide to complete the questionnaire.**

*In this research study we will use the information you give. We will only use information that we need for the research study. Everyone involved in this study will keep your data safe and secure. We will also follow all privacy rules. At the end of the study we will save some of the data in case we need to check it. We will make sure no-one can work out who you are from the reports we write.*

**I AGREE I AM ELIGIBLE AND AGREE TO TAKE PART IN THIS RESEARCH.**

**Please tick.**

**YES**

**NO - Thank you for your time.**

*If YES:*

Is someone else is helping you to complete this questionnaire?

YES___________________________________

NO

Is someone translating this questionnaire for you?

*If yes please state which language.*

YES LANGUAGE? ______________

NO

Section 2: These questions ask some background information about you

1. What sex were you assigned at birth?

Male

Female

Prefer not to say

1. What is your age in years? ________________________(leave blank if you prefer not to say)
2. What is your postcode? ______________________ (leave blank if you prefer not to say)
3. What is your country of birth?______________________ (leave blank if you prefer not to say)
4. Which of the following best describes your ethnicity?

White:- British / White:- Irish / White:- Other White background

Mixed:- White and Black Caribbean / Mixed:- White and Black African / Mixed:- White and Asian / Mixed:- Other mixed background

Black or Black British:- African / Black or Black British:- Caribbean / Black or Black British:- Any other Black background

Asian or Asian British:- Indian / Asian or Asian British:- Pakistani / Asian or Asian British:-Bangladeshi / Asian or Asian British:- Other Asian background

Chinese

Other ethnic group not represented by these options __________________________

Prefer not to say

1. What religion are you?

Christian

Buddhist

Hindu

Muslim

Sikh

Jewish

Other

No religion

Prefer not to say

1. What is the highest level of education you have completed?

Primary school

Secondary school up to 16 years

Higher or secondary or further education (A-levels, BTEC, etc.)

College or university

Post-graduate degree

Prefer not to say

1. Which of the following describes you best?

I am raising my child(ren) with a partner

I am a single parent

Other

Prefer not to say

1. Please tick all relatives that live with you in the same house:

Spouse and/or cohabiting partner

Son and/or daughter (incl. adopted, foster or step-child/children)

Son-in-law/daughter-in-law

Parent/guardian

Parent-in-law

Brother/sister (incl. adopted, step or foster siblings)

Brother/sister-in-law

Grand-child

Grand-parent

Other relative/living situation________________________

1. How many children do you have or are the guardian of?

1

2

3

4 or more

1. How many children do you have or are the guardian of **aged under 5 years old?**

1

2

3

4 or more

Section 3: These questions ask about routine vaccines recommended to all children in the UK by NHS General Practitioners (GPs) and other NHS staff such as health visitors & practice nurses.

#### (The routine vaccines as recommended in The Red Book you are given when your baby is born)

**Please answer how much you agree or disagree with the following statements by circling your response.**

1. It is important for parents to get the recommended vaccines for their child/children.

Strongly agree|Agree|Somewhat agree|Neither agree nor disagree|Somewhat disagree|Disagree|Strongly disagree|

Not applicable__________________

1. Most parents I know in my community have their child/children vaccinated with all the recommended vaccines

Strongly agree|Agree|Somewhat agree|Neither agree nor disagree|Somewhat disagree|Disagree|Strongly disagree|

Not applicable_________________

1. Some people choose not to get their child/children vaccinated because of religious and/or cultural reasons.

Strongly agree|Agree|Somewhat agree|Neither agree nor disagree|Somewhat disagree|Disagree|Strongly disagree|

Not applicable___________________

1. Wider family members such as grandparents, influence whether parents decide to get their child vaccinated.

Strongly agree|Agree|Somewhat agree|Neither agree nor disagree|Somewhat disagree|Disagree|Strongly disagree|

Not applicable___________________

1. Have you personally ever refused or delayed any vaccines your child/children was/were offered before age 5 years?

No – my child/children have had all their vaccines when offered.

Yes – my child/children have not had or been vaccinated later than offered.

*If yes:*

1. Which vaccines did your child/children not have or have later?

_______________________________________________________________

1. Which of the following is most true for you?

Child/children went onto have their vaccine later

I plan to have my child/children vaccinated later

I am not sure whether I will have my child/children vaccinated later

I have no plans to have my child/children vaccinated later

I will refuse to have my child/children vaccinated later

1. Is there anything else you would like to add about your opinions on vaccines recommended for children in the UK?

**__________________________________________________________________________________**

Section 4: Information and access to your child/children’s vaccines before they are aged 5 years

**Please answer how much you agree or disagree with the following statements about information and booking your child/children’s vaccine appointments before aged 5 years by circling your response.**

1. I was given enough information by healthcare professionals (doctors, midwives, health visitors, nurses and others) before my child/children vaccine appointments.

Strongly agree|Agree|Somewhat agree|Neither agree nor disagree|Somewhat disagree|Disagree|Strongly disagree|

Not/applicable___________________

1. I understood the information that I was given before my child/children’s appointments by healthcare professionals (doctors, midwives, health visitors, nurses and others).

Strongly agree|Agree|Somewhat agree|Neither agree nor disagree|Somewhat disagree|Disagree|Strongly disagree|

Not/applicable___________________

1. I knew how to book my child/children’s vaccine appointments.

Strongly agree|Agree|Somewhat agree|Neither agree nor disagree|Somewhat disagree|Disagree|Strongly disagree|

Not/applicable___________________

1. I was given enough opportunity to ask questions before my child/children’s vaccine appointments.

Strongly agree|Agree|Somewhat agree|Neither agree nor disagree|Somewhat disagree|Disagree|Strongly disagree|

Not/applicable___________________

1. I would have preferred the information provided by healthcare professionals about vaccines to be interpreted by a family member or friend into my own language before my child/children’s vaccine appointments.

Strongly agree|Agree|Somewhat agree|Neither agree nor disagree|Somewhat disagree|Disagree|Strongly disagree|

Not/applicable___________________

1. Before my child/children’s vaccine appointment I felt confident about getting my child/children vaccinated.

Strongly agree|Agree|Somewhat agree|Neither agree nor disagree|Somewhat disagree|Disagree|Strongly disagree|

Not/applicable___________________

1. I found it easy to book my child/children’s vaccine appointments at their GP surgery at a time that was convenient for me and my family.

Strongly agree|Agree|Somewhat agree|Neither agree nor disagree|Somewhat disagree|Disagree|Strongly disagree|

Not/applicable___________________

1. I found it easy to travel to my GP surgery for my child/children’s vaccine appointments.

Strongly agree|Agree|Somewhat agree|Neither agree nor disagree|Somewhat disagree|Disagree|Strongly disagree|

Not/applicable___________________

1. I found it difficult to take my child/children to my GP surgery for their vaccine appointments due to work.

Strongly agree|Agree|Somewhat agree|Neither agree nor disagree|Somewhat disagree|Disagree|Strongly disagree|

Not/applicable___________________

1. I found it difficult to take my child/children to my GP surgery for their vaccine appointments due to my caring responsibilities (other children, relatives etc).

Strongly agree|Agree|Somewhat agree|Neither agree nor disagree|Somewhat disagree|Disagree|Strongly disagree|

Not/applicable___________________

1. I found it difficult to take my child/children to my GP surgery for their vaccine appointments due to religious and cultural events such as Ramadan, weddings, funerals.

Strongly agree|Agree|Somewhat agree|Neither agree nor disagree|Somewhat disagree|Disagree|Strongly disagree|

Not/applicable___________________

1. I found it difficult to take my child/children to my GP surgery for their vaccine appointments due to having been away visiting family overseas.

Strongly agree|Agree|Somewhat agree|Neither agree nor disagree|Somewhat disagree|Disagree|Strongly disagree|

Not/applicable___________________

1. I felt comfortable taking my child/children for vaccine appointments at their GP surgery.

Strongly agree|Agree|Somewhat agree|Neither agree nor disagree|Somewhat disagree|Disagree|Strongly disagree|

Not/applicable___________________

1. I felt welcome taking my child/children for vaccine appointments at their GP surgery.

Strongly agree|Agree|Somewhat agree|Neither agree nor disagree|Somewhat disagree|Disagree|Strongly disagree|

Not/applicable___________________

1. Appointments at other locations such as children’s centres, community centres, nurseries would make it easier for me to take my child/children to be vaccinated.

Strongly agree|Agree|Somewhat agree|Neither agree nor disagree|Somewhat disagree|Disagree|Strongly disagree|

Not/applicable___________________

1. Drop-in clinics (no fixed appointments) would make it easier to take my child/children to be vaccinated.

Strongly agree|Agree|Somewhat agree|Neither agree nor disagree|Somewhat disagree|Disagree|Strongly disagree|

Not/applicable___________________

1. More appointments after school hours would make it easier to take my child/children to be vaccinated.

Strongly agree|Agree|Somewhat agree|Neither agree nor disagree|Somewhat disagree|Disagree|Strongly disagree|

Not/applicable___________________

1. I know that if I miss a vaccine appointment for my child/children I am able to rebook and get my child/children vaccinated later.

Strongly agree|Agree|Somewhat agree|Neither agree nor disagree|Somewhat disagree|Disagree|Strongly disagree|

Not/applicable___________________

Section 5: Your child/children’s vaccine appointments and COVID-19 disease

Please answer how much you agree or disagree with the following statements about vaccines routinely recommended to all children in the UK by circling your response.

(NOT COVID-19 vaccines for children)

1. The Covid-19 pandemic has made me think it is more important for children to have their routine vaccines recommended to all children in the UK.

Strongly agree|Agree|Somewhat agree|Neither agree nor disagree|Somewhat disagree|Disagree|Strongly disagree|

Not/applicable___________________

1. The Covid-19 pandemic has made me question whether the vaccines routinely recommended to all children are safe.

Strongly agree|Agree|Somewhat agree|Neither agree nor disagree|Somewhat disagree|Disagree|Strongly disagree|

Not/applicable___________________

1. The Covid-19 pandemic has made me more likely to refuse or delay vaccines routinely recommended by for my child/children in the UK.

Strongly agree|Agree|Somewhat agree|Neither agree nor disagree|Somewhat disagree|Disagree|Strongly disagree|

Not/applicable___________________

1. I have delayed or refused a vaccine routinely recommended for my child/children since the start of the Covid-19 pandemic.

Strongly agree|Agree|Somewhat agree|Neither agree nor disagree|Somewhat disagree|Disagree|Strongly disagree|

Not/applicable___________________

1. The Covid-19 pandemic has increased my knowledge about all vaccines including vaccines routinely recommended for children.

Strongly agree|Agree|Somewhat agree|Neither agree nor disagree|Somewhat disagree|Disagree|Strongly disagree|

Not/applicable___________________

1. The Covid-19 pandemic means I have more questions about all vaccines including the routinely recommended vaccines for children.

Strongly agree|Agree|Somewhat agree|Neither agree nor disagree|Somewhat disagree|Disagree|Strongly disagree|

Not/applicable___________________

Section 6: Any other comments.

Are there any other comments you want to make about why you think some children may not be vaccinated or vaccinated late?

**__________________________________________________________________________________**

**__________________________________________________________________________________**

**__________________________________________________________________________________**

Section 7: Thank you

Thank you for completing this questionnaire about childhood vaccination.

For this research study we are planning to combine the results of this questionnaire with the findings from focus groups with parents of children aged under 10 years. Focus groups will be held online or if you live close enough in the Church Street area of Westminster, North-West London.

If you would like to help us further and would be willing to participate in an approximately 45-60min focus group discussing similar topics as covered in this questionnaire please tick yes.

**YES – Please complete section 8 on the next page**

**NO - Thank you for your time.**

Section 8: Focus group interest

Please provide, your email address or mobile phone details below. You will be provided with a £10 Tesco gift voucher to thank you for your participation in the focus groups.

Alternatively, you can contact me directly on: [h.skirrow@imperial.ac.uk](mailto:h.skirrow@imperial.ac.uk)

- Email:
- Phone Number:

This research is part Dr Helen Skirrow’s PhD into missed and late childhood vaccination in the UK which is funded by the National Institute of Health Research, doctoral research fellowship award number NIHR300907.

# Supporting 2: Focus Group Topic Guide

Focus Group Topic Guide Why did nobody ask us?!

*Understanding why parents and families think some children get vaccinated late or miss out on some of their routine recommended childhood vaccines.*

1. Can we start of by talking about how many children we have and how old they are?
2. Can you tell me about your thoughts on vaccinations?
   1. What do you see as the benefits of vaccinations for children?
   2. Do you have any concerns (if any) about vaccinations for children?
3. We know that some children miss getting their routine vaccines before the age of 5 years or are late being vaccinated?
   1. Why do you think this could be?
      1. Do you know of parents/families getting their children vaccinated abroad? Or do you think being abroad when their vaccines are due might be a reason vaccinations are missed?
      2. Do you know of parents/families paying to have their children vaccinated privately? Do you know why this might be?
      3. Do you think religious or cultural events such as Ramadan or family weddings might mean vaccinations are missed?
4. How confident were you in your decision to have your child/ren vaccinated?
   1. Were you given enough information on vaccines recommended before age 5 years?
   2. Are you given enough time and opportunity to ask questions about vaccines due before 5 years?
   3. What did you think of the of the information provided by the NHS/GP surgery about vaccines? How could information be improved? Interpreted by a family member or friend? Information leaflets in different languages? Formats? When information was availabile?
   4. Did you have any concerns about the pain of vaccination or needles?
   5. Do you think your experience of getting vaccinated in pregnancy (if you did) influenced you?
5. What has been your experience of getting your children vaccinated at your GP surgery?
6. Did you get reminders from your GP about your child/ren’s vaccine appointments?
7. Did your GP surgery offer appointments at convenient times?
8. How do you find the process of booking appointments?
9. Has Covid-19 changed your views on routine childhood vaccines?
   1. Has it made you more or less trusting?
   2. Do you have more or less questions?
10. How do you think we can improve vaccine services so more children can be vaccinated and also vaccinated on time?
    1. Do you think vaccinations being offered at different locations such as nurseries or children centres or when you take your child to a different hospital appointment might help?
11. Any other comments you want to make?

# Supporting 3: Number of freetext responses to questionnaire

- Paper respondents: 41/42 answered the section about Covid-19 and children’s vaccinations.
- Online respondents to the Covid-19 questions varied between 353 and 374 responses depending on the question.
- Online respondents - 16 made free-text comments in the Covid-19 section and there were 52 online comments made in the other free-text sections of the questionnaire that directly referenced the Covid-19 pandemic and routine childhood vaccinations.
- Only one paper respondent left a comment referencing the pandemic and their children’s routine vaccinations.

# Supporting 4: Covid-19 and knowledge about routine vaccines recommended for children.

Total respondents answering question n=412

# Supporting 5: Impact of Covid-19 on parents’ attitudes to routine childhood vaccinations by Ethnicity

| Chi square analysis of parents’ responses to Covid-19 questions dichotomised into ethnicity groups. | | | |
| --- | --- | --- | --- |
| *The Covid-19 pandemic has increased my knowledge about all vaccines including vaccines routinely recommended for children.* | | | |
|  | **Agreed***  **n (%)** | **Disagreed****  **n (%)** | **p value** |
| *White British, White Irish, Other White background* | 137 (49) | 141 (51) | 0.163 |
| *Mixed, Black or Black British, Asian or Asian British, Chinese or other or unknown Ethnicity group* | 65 (57) | 49 (43) |  |
| *The Covid-19 pandemic means I have more questions about all vaccines including the routinely recommended vaccines for children.* | | | |
| *White British, White Irish, Other White background* | 84 (30) | 197 (70) | **<0.0001** |
| *Mixed, Black or Black British, Asian or Asian British, Chinese or other or unknown Ethnicity group* | 71 (59) | 49 (41) |  |
| *The Covid-19 pandemic has made me think it is more important for children to have their routine vaccines recommended to all children in the UK.* | | | |
| *White British, White Irish, Other White background* | 167 (62) | 104 (38) | 0.7900 |
| *Mixed, Black or Black British, Asian or Asian British, Chinese or other or unknown Ethnicity group* | 75 (63) | 44 (37) |  |
| *The Covid-19 pandemic has made me more likely to refuse or delay vaccines routinely recommended by for my child/children in the UK.* | | | |
| *White British, White Irish, Other White background* | 22 (8) | 258 (92) | **<0.0001** |
| *Mixed, Black or Black British, Asian or Asian British, Chinese or other or unknown Ethnicity group* | 30 (25) | 90 (75) |  |
| *I have delayed or refused a vaccine routinely recommended for my child/children since the start of the Covid-19 pandemic.* | | | |
| *White British, White Irish, Other White background* | 29 (11) | 240 (89) | **0.0031** |
| *Mixed, Black or Black British, Asian or Asian British, Chinese or other or unknown Ethnicity group* | 26 (22) | 91 (78) |  |
| *It is important for parents to get the recommended vaccines for their child/children.* | | | |
| *White British, White Irish, Other White background* | 301 (97) | 8 (3) | **0.0017** |
| *Mixed, Black or Black British, Asian or Asian British, Chinese or other or unknown Ethnicity group* | 119 (89) | 15 (11) |  |
| *The Covid-19 pandemic has made me question whether the vaccines routinely recommended to all children are safe.* | | | |
| *White British, White Irish, Other White background* | 50 (18) | 230 (82) | **<0.0001** |
| *Mixed, Black or Black British, Asian or Asian British, Chinese or other or unknown Ethnicity group* | 61 (51) | 59 (49) |  |

*Respondents were first dichotomised into those that answered the question: Which of the following best describes your ethnicity? as ‘White, White Irish or Other White background’ or those that answered any of ‘Any Mixed background, Black or Black British or any Black background, Asian or British Asian or any Asian background, Chinese or other or unknown ethnicity group’.*

*Likert responses to the questions on how their attitudes to routine childhood vaccines had been impacted by the Covid-19 pandemic were then also dichotomised into *Agreed (Strongly agree, Agree or Somewhat Agree) and **Disagreed (Neither agree or disagree, somewhat disagree, Disagreed or Strongly Disagree).*

*P values reported are from Chi Square test results.*

# Supporting 6: Covid-19 pandemic and the importance of routine vaccines

*Total respondents answering question n=405.*

# Supporting 7: Covid-19 pandemic and likelihood of refusing or delaying routine vaccines.

*Total respondents answering question n=414.*

# Supporting 8: Covid-19 pandemic and having refused or delayed routine vaccines.

*Total respondents answering question n=394.*

# Supporting 9: Importance of getting children vaccinated.

*Total respondents answering question* n=459

# Supporting 10: Covid-19 pandemic and safety of routine vaccines

*Total respondents answering question n=415.*
